# Factors associated with uptake of post-abortion family planning in Shire town, Tigray, Ethiopia

**DOI:** 10.1186/s13104-018-4029-7

**Published:** 2018-12-27

**Authors:** Yohannes Moges, Tesfay Hailu, Balem Dimtsu, Zemenu Yohannes, Bayew Kelkay

**Affiliations:** 1 Department of Midwifery, College of Health Sciences, Debre Birhane University, Debre Birhane, Ethiopia; 2 Department of Midwifery, College of Health Sciences, Aksum University, Axum, Ethiopia; 3 Department of Midwifery, College of Health Sciences, Mekelle University, Mekelle, Ethiopia; 4 Department of Midwifery, College of Medicine and Health Sciences, Hawassa University, Awasa, Ethiopia; 5 School of Midwifery, College of Medicine and Health Sciences, University of Gondar, Gondar, Ethiopia

**Keywords:** Post-abortion, Contraceptive utilization, Ethiopia

## Abstract

**Objectives:**

Post-abortion contraceptive service is pivotal for the prevention of unwanted pregnancy and alleviation of its complication. Worldwide half of the pregnancy is unplanned, whereas unwanted pregnancy ends up with abortion. This study assessed post-abortion contraceptive uptake and associated factors among abortion service users at health institution in Shire town, North Ethiopia. Institutional based cross-sectional study was conducted from December 15/2016 to March 15, 2017, in Shire town. Data were collected using systematic random sampling technique. Bivariate and multivariable analyses were done to determine the association of each independent variable with the dependent variable.

**Results:**

Overall post-abortion contraceptive utilization in this study was 61.5%. Married [AOR 2.59, 95% CI (1.16, 5.65)], completed College education [AOR 5.69, 95% CI (1.61, 20.11)], previous contraceptive used [AOR 3.62, 95% CI (1.77, 7.40)], counseling of family planning [AOR 3.53 95% CI (1.69, 7.37)], grand multipara [AOR 7.91, 95% CI (1.66, 37.74)] and public health institution [AOR 5.95, 95% CI (3.03, 11.72)] were significantly associated with the post-abortion contraceptive utilization. In this study, post-abortion contraceptive utilization was about two-third. Being married, had been completing a college education, had been receiving family planning counseling, previous contraceptive usage, abortion care service at public health institution, and being grand multiparty were determinants of post-abortion contraceptive utilization.

## Introduction

According to the report of 2012, around 213 million pregnancies occurred worldwide, of which 89% occurred in the developing world. Out of the total, 85 million pregnancies were unintended; of these, 50% ended in abortion [1]. In 2013, 43,684 women lost their lives as a result of complications from abortion worldwide [2]. The WHO report indicated that every year an estimated 22,000 women died in developing countries from abortion complication [3]. Around 8.3 million African and 2.7 million eastern African women have induced abortions every year. Consequently, in Africa, 9% of maternal death was due to an unsafe abortion [4].

Worldwide, due to non-use of contraception, more than 40% of pregnancies are unplanned. Unmet need for family planning is the leading cause of unintended pregnancy. It is the frontline cause of induced abortion. Three out of four unsafe abortions could be avoided if family planning need was fully met [5]. In addition to, the contraceptive is a major primary prevention strategy for unwanted pregnancies, which contraceptive reduces about 230 million births every year across the globe [6].

In Ethiopia, every year 620,296 induced abortions were performed, and 103,648 women were treated for complications of abortions. Nationally, the annual abortion ratio is 17.6/100 live births, and 38% of pregnancies were unplanned [7].

In sub-Saharan Africa, 23% were utilized any types of family planning, and 18% of modern family planning methods. The contraceptive prevalence rate in Ethiopia was 36% for all women and 42% for currently married women [8, 9].

Post-abortion family planning program shall be offered to all women, regardless of their age, marital status. Providing information and initiation of a method are the only first steps in preventing unwanted pregnancy and subsequent unsafe abortions. When counseling and services are offered immediately after treatment, acceptance of contraceptive is high, reducing costs and optimizing staff time [10].

Studies recommended post-abortion contraceptive service shall be given to prevent unwanted pregnancy and abortion-related complication. To achieve the Sustainable Development Goal targets in relation to maternal health, by the Ministry of Health of Ethiopia post-abortion contraceptive service is crucial. Hence, this study assessed post-abortion contraceptive uptake and associated factors among abortion service users at health institution in Shire town, North Ethiopia. The findings of this study are expected to be a useful input to the local health authorities, stakeholders, health care providers, and the community.

## Main text

### Study area and period

This study was conducted in public health institutions from December 15/2016 to March 15, 2017, in Shire town. Shire is the administrative town of the North-western Zone and is located at 1094 km away from the capital city of Ethiopia, Addis Ababa and 301 km from Mekelle, which is the administrative city of the Tigray region.

### Study design

An institution based cross-sectional study was conducted.

#### Sample size determination

The sample size was determined by using a single population proportion. The utilization of post-abortion contraceptive in Debre Marcos town was 59.2% in 2014 [11]. For determining the sample size of this study, 5% level of significance (a = 0.05), 5% margin of error (d = 0.05) and 10% non-responsive rate were taken into account. Based on this, the total sample of the study was determined to be 408.

#### Sampling procedure

All health institutions at Shire town which provided abortion service were included in the study. From all those health institutions, on average, there were 823 (N) abortion service users per 3 months. Then, the possible number of respondents in each of the health institutions of the study area were allocated proportionally based on the 3 months an average number of client flow for abortion services. After this, the skip interval was calculated in each institution for selecting the respondents systematically as K = N/n, where K is the skipping interval and was 2. Hence, for this study, a systematic sampling method was applied. The starting point was selected randomly and then beginning from the starting point, each individual was selected that correspond to the skip interval.

### Data collection instruments and techniques

Data were collected using structured and pre-tested questioners. The questionnaires were first prepared in English, and translated into the local language (Tigrigna). An exit interview was conducted where questions and answers could not be overheard.

### Data processing and analysis

The data entered using EP info and analyzed using SPSS version 20 software. Descriptive statistics carried out for frequencies. Bivariate analysis from analytical statistics was applied and variables showing *p* value < 0.05 were taken for multivariable. Odds ratio with their 95% confidence intervals were computed to identify the presence of association and statistical significance is p < 0.05.

### Socio-demographic characteristics

A total of 400 abortion services users were interviewed with a response rate of 98%. From the total participants, 287 (71.8%) came from urban. Whereas 153 (38.2%) were in the age group of 20–24 followed by 15–19, 76 (19%) (Table 1).

**Table 1:** Socio-demographic characteristic of study participants of Shire town, Tigray Regional State, Ethiopia 2017 (n = 400)

Variables	Frequency (n)	Percentage (%)
*Age*		
15–19	76	19.0
20–24	153	38.2
25–29	70	17.5
30–34	59	14.8
35+	42	10.5
*Religion*		
Orthodox	337	84.3
Muslim	41	10.3
Catholic	11	2.7
Protestant	11	2.7
*Marital status*		
Unmarried	183	45.8
Married	188	47.0
Widowed	9	2.2
Divorced	20	5.0
*Educational status*		
No formal education	77	19.3
Elementary [1–8]	102	25.5
Secondary [9–12]	164	41.0
Completed college	57	14.2
*Occupation*		
Student	93	23.3
Farmer	47	11.7
Merchant	35	8.7
Employed	127	31.8
House maids	59	14.7
Unemployed/job seeker	39	9.8
*Monthly income*		
≤ 500	15	3.8
501–1000	151	37.7
> 1000	234	58.5

#### Reproductive history

The study finding revealed that 257 (64.3%) of respondents had a history of the previous pregnancy. Among those, 65 (16.3%) participants had also a history of abortion. Majority of the Participants (39.3%) were nulliparous mothers. Of all the respondents, 212 (53%) had 1–3 alive children during the study period (Table 2).

**Table 2:** Reproductive history of study participants of Shire town, Tigray Regional State, Ethiopia, 2017 (n = 400)

Variable	Frequency (n)	Percentage (%)
*Parity*		
0	157	39.3
1	143	35.7
2–4	82	20.5
≥ 5	18	4.5
*Number of alive children*		
0	158	39.5
1–3	212	53.0
≥ 4	30	7.5
*Types of abortion*		
Induced	325	81.2
Spontaneous	75	18.8
*Condition of the pregnancy*		
Planned/wanted	39	9.7
Unplanned but wanted	75	18.8
Unplanned/unwanted	286	71.5
*Reason for termination*		
Rape	116	29.0
Incent	118	29.5
Maternal indication	60	15.0
Fetal deformity	31	7.7
Spontaneously	75	18.8
*Methods used to terminate the current pregnancy*		
Medication abortion	195	48.8
Manual vacuum aspiration	116	29.0
Mixed procedure	89	22.2

Most of the study participants (71.5%) reported that the current pregnancy was unwanted. The majority of participants (81.2%) terminated their pregnancy intentionally. From the total study participants, 195 (48.8%) respondents’ pregnancy was terminated by medication. Of the participants (69.8%) were reported that their pregnancy was terminated at 1st trimester (Table 2).

#### Contraceptive and health institution related information

Of the total, 236 respondents (59%) reported that they used at least one form of contraceptive previously. From those who had used contraceptive in the past, 182 respondents (77.1%) were developed the side effect while they used the contraceptive, as reported by respondents. Among the respondents (67.2%) got post-abortion contraceptive counseling after receiving abortion care services.

#### Utilization of post-abortion contraceptive

From the total, 246 respondents (61.5%) were utilized contraceptive after they got post-abortion care services. Among those who used the contraceptive, 51.3% and 31.7% of the participants were used implants and injection, respectively. Only 10 of the clients (4%) chose IUCD. From those who utilized contraceptives (66.3%) adopted contraceptive after they got abortion services in public health institutions.

Had not received contraceptive counseling (26%), wished to give birth soon (22.8%), fear of side effect (18.8%), since the current pregnancy is raped (11.7%), male partner refusal (9%), and preferring natural FP (7.2%) were the most common reasons cited by respondents who did not use post-abortion contraceptive care services.

#### Factors associated with post-abortion contraceptive utilization

In multivariable analysis, married women were 2.5 times more likely to utilize contraceptive as compared to unmarried women [AOR 2.59, 95% CI (1.16, 5.65)]. The odds of using contraceptive was higher among women who completed college education than those who had no formal education [AOR 5.69, 95% CI (1.61, 20.11)]. Similarly, women who had the previous history of contraceptive usage were 3.6 times more likely to utilize contraceptive as compared to their counterpart [AOR 3.62, 95% CI (1.77, 7.40)].

Women who had received family planning counseling were 3.5 times more likely to utilize contraceptive as compared to women who did not obtain the counseling [AOR 3.53, 95% CI (1.69, 7.37)]. Women who got abortion services at public health institutions were 5.9 times more likely to utilize contraceptive as compared to those who got abortion care services at private clinics [AOR 5.95, 95% CI (3.03, 11.72)]. In addition, the odds of using contraceptive was higher among grand multipara than nulliparous women [AOR 7.91, 95% CI (1.66, 37.74)] (Table 3).

**Table 3:** Factors associated with post abortion contraceptive utilization among abortion services user in Shire town health institution, Tigray, Ethiopia, 2016 (n = 400)

Variables	Post-abortion contraceptive utilization		Unadjusted and adjusted OR	
	Yes	No	COR (95% CI)	AOR (95% CI)
*Marital status*				
Married	157	31	7.46 (4.59, 12.12)*	2.59 (1.16, 5.65)*
Widowed	3	6	0.74 (0.18, 3.04)	0.71 (0.11, 4.72)
Divorced	12	8	2.21 (0.86, 5.67)	0.87 (0.19, 3.85)
Unmarried	74	109	1	1
*Educational status*				
Elementary	33	69	0.94 (0.500, 1.76)	0.91 (0.34, 2.39)
Secondary	138	26	10.41 (5.54, 19.58)	6.55 (2.42, 17.77)
Completed college	49	8	12.01 (4.96, 29.09)*	5.69 (1.61, 20.11)*
No formal education	26	51	1	1
*Parity*				
1	130	13	19.07 (9.87, 36.85)	4.80 (1.94, 11.89)
2–4	55	27	3.89 (2.206, 6.84)*	2.06 (0.82, 5.17)
≥ 5	7	11	1.21 (0.45, 3.31)	7.91 (1.66, 37.74)*
0	54	103	1	1
*Have you ever used contraceptives*				
Yes	192	44	8.89 (5.60, 14.11)*	3.62 (1.77, 7.40)*
No	54	110	1	1
*Have you counseled on contraceptives*				
Yes	203	66	6.29 (3.98, 9.96)*	3.53 (1.69, 7.37)*
No	43	88	*1*	
*Ownership of the facility*				
Public	163	35	6.68 (4.21, 10.58)*	5.95 (3.03, 11.72)*
Private	83	119	*1*	1

* Significant at p-value < 0.05

### Discussion

In this study, the magnitude of post-abortion contraceptive utilization was 61.5%. This finding agreed with the studies conducted in Debre Markos (59.2%) and Addis Ababa (57%) [11, 12] and it was lower than the studies conducted in Southern Ethiopia (83%), Tanzania (89%), Pakistan (72.9%), and Brazil (97.4%) [13–16]. On the other hand, it was higher than the studies conducted in Dessie (47.5%) and Nepal (49.5%) [17, 18]. This variation of contraceptive utilization could be due to the respondents’ level of awareness, educational level, religious beliefs and various misconceptions about contraceptive and family planning services deference among study settings.

Married women were 2.5 times more likely to utilize post-abortion contraceptive as compared to unmarried women. The current study was in line with the study conducted in Jimma that was 6.7 times more likely to adopt post abortion family planning than unmarried women [19]. However, the study conducted in Debre Markos showed that the married women who used post-abortion contraceptive were 44% less likely to use contraceptive compared to unmarried women [11]. The possible reason could be that the married women would live together with their husbands so that there might be influence from their partners.

The odds of using contraceptive were higher among women who completed college education than those who had no formal education. This finding was supported by studies conducted in Addis Ababa, Debre Markos, Pakistan and Tanzania [11–13, 15]. This could be educated women are more eager to access information about reproductive health issues which again enable them to pass informed decisions. Furthermore, educated women’s are more concerned about their carrier development and they would put their child desire aside.

Women who had the previous history of contraceptive usage were 3.6 times more likely to utilize contraceptive as compared to their counterpart. Similarly, the study conducted in Addis Ababa, Dessie, and Pakistan showed that the previous history of contraceptive usage was found to be significantly associated with post-abortion contraceptive utilization [12, 13, 18]. The possible explanation could be the previous exposure to family planning services might influence the awareness of women towards post-abortion contraceptive utilization.

In this study, family planning counseling was found to be significantly associated with post-abortion contraceptive utilization. This finding was in line with the study conducted in Debre Markos where women who received family planning counseling were 4 times more likely to utilize contraceptives [11]. This showed that post-abortion period is the right time to introduce contraceptive advice because women are more ready to receive messages.

In this finding, an abortion care service at public health institution was found to be significantly associated with post-abortion contraceptive utilization. In contrary, had been receiving abortion care in a private clinic was two times more likely to have been utilized post-abortion contraceptive [12]. This is due to that currently, in Ethiopia family planning services are provided free of charge in all public health facilities whereas the services in private facilities had cost. The odds of using contraceptive were higher among grand multipara than nulliparous women. The current study was in line with studies conducted in Nepal and Addis Ababa [12, 17]. This could be due to that grand multiparous mothers might want to limit their number of children.

### Conclusion

In this study, post-abortion contraceptive utilization was low. Being married, had been completing a college education, had been receiving family planning counseling, previous contraceptive usage, abortion care service at a public health institution, and being grand multiparty were a statistically significant association with the post-abortion contraceptive.

### Recommendation

Private facilities should strengthen the family planning services that will help to increase post-abortion contraceptive utilization. Health care providers shall provide counseling on time following abortion before the women left the facility. Much should be done by obstetric care providers to strength the post-abortion contraceptive counseling and increase contraceptive utilization after abortion. In addition, due attention should be given for improving maternal education.

#### Limitations

Although the study was conducted in all types of health facilities, it might not give the real utilization of post-abortion contraceptive because the information was no gathered whether the women used the contraception in other health facilities or not after they discharged, which was the limitation of this study.

## Abbreviations

AOR: adjusted odds ratio
IUCD: Intra Uterine Contraceptive Device

## References

[1] Sedgh G, Singh S, Hussain R (2014). Intended and unintended pregnancies worldwide in 2012 and recent trends. Stud Fam Plann.

[2] Kassebaum NJ, Coggeshall MS, Shackelford KA, Steiner C, Heuton KR (2014). Global, regional, and national levels and causes of maternal mortality during 1990–2013: a systematic analysis for the Global Burden of Disease Study 2013. Lancet.

[3] WHO (2014). Key facts on induced abortion worldwide.

[4] Institute G, Lane M, New York U. Incidence and trends of abortion in Africa. 2014. p. 1–2.

[5] WHO (2008). Global and regional estimates of the incidence of unsafe abortion and associated mortality in 2008.

[6] Haddad LB, Nour NM (2009). Unsafe abortion: unnecessary maternal mortality. Rev Obstetr Gynecol.

[7] Moore AM, Gebrehiwot Y, Fetters T (2016). The estimated incidence of induced abortion in Ethiopia, 2014: changes in the provision of services since 2008. Int Perspect Sex Reprod Health..

[8] WHO (2008). Safe abortion: technical and policy guidance for health systems.

[9] Central Statistical Agency (CSA) [Ethiopia] and ICF (2016). Ethiopia demographic and health survey 2016.

[10] WHO (2013). Post-abortion family planning: a practical guide for programme managers.

[11] Kokeb L, Admassu E, Kassa H, Seyoum T (2015). Utilization of post abortion contraceptive and associated factors among women who came for abortion service: a hospital-based cross-sectional study. J Fam Med Dis Prev.

[12] Prata N, Bell S, Holston M, Gerdts C, Melkamu Y (2011). Factors associated with choice of post-abortion contraception in Addis Ababa, Ethiopia. Afr J Reprod Health..

[13] Azmat SK, Hameed W, Ishaque M, Mustafa G, Ahmed A (2011). Post-abortion care family planning use. Pak J Public Health..

[14] Ferreira ALC, Souza AI, Lima RA, Braga C (2008). choices on contraceptive methods in post-abortion family planning clinic. Reproduct Health.

[15] Rasch V, Yambesi F, Massawe S (2008). Medium and long-term adherence to postabortion contraception among women having experienced unsafe abortion. BMC Pregnancy Childbirth..

[16] Samuel M, Fetters T, Desta D (2016). Strengthening post-abortion family planning services in Ethiopia: expanding contraceptive choice and improving access to long-acting reversible contraception. Glob Health Sci Pract..

[17] Shrestha S (2012). Post abortion choice and acceptance of contraception. Nepal J Obstetric Gynaecol.

[18] Seid A, Gebremariam A, Abera M (2010). Integration of family planning services within post abortion care at health facilities in Dessie-North East Ethiopia. Technol Arts Res J..

[19] Erko EK, Abera M, Admassu B (2016). Safe abortion care, utilization of post abortion contraception and associated factors, Jimma Ethiopia. J Women’s Health Care..

